# Importance of local and systemic factors in preventing implant displacement in the mandibular body: a scoping review of existing literature

**DOI:** 10.1186/s12903-024-04671-2

**Published:** 2024-08-01

**Authors:** José Rosas-Díaz, Maria Eugenia Guerrero, Diana Castillo-Andamayo, Maisely Galindo-Gómez, Marco García-Luna, Luis Cervantes-Ganoza, César Cayo-Rojas

**Affiliations:** 1 School of Stomatology, Universidad Privada San Juan Bautista, Jose Antonio Lavalle Avenue 302- 304 (Ex Hacienda Villa), Chorrillos, Lima, Peru; 2 Faculty of Stomatology, Universidad Peruana Cayetano Heredia, Lima, Peru; 3 Medico Surgical Department, Faculty of Dentistry, Universidad Nacional Mayor de San Marcos, Lima, Peru; 4 Faculty of Stomatology, Universidad Inca Garcilaso de la Vega, Lima, Peru

**Keywords:** Dental implants, Displacement, Mandible, Intraoperative complications

## Abstract

**Background:**

The aim of this research was to analyse the current literature on displaced dental implants in the mandibular body, including local and systemic variables related to their cause, and to identify the most frequent location.

**Methods:**

The study conducted a search of three databases (Pubmed, Scopus, and Web of Science) using specific index terms such as ‘dental implant’, ‘displacement’, ‘dislocation’, ‘displaced’, and ‘mandible’. The analysis focused on the direction of displacement and the characteristics of the bone tissue (bone quality, density, and quantity) in cases where dental implants were displaced.

**Results:**

A total of 371 articles were obtained. Thirteen of these articles were selected and read in full. To define bone quality, the Lekholm and Zarb classification, modified by Rosas et al., was used. The type II-B bone, which is characterized by thick cortical bone surrounding cancellous bone with extremely wide medullary spaces, presented the largest number of complications. Twenty-two cases were found in which the displacement direction was horizontal. Of these, four were displaced vestibularly, fourteen lingually, and four remained in the center. Additionally, 24 cases presented vertical displacement, with 12 displaced towards the inferior border of the mandible, 9 towards the middle or adjacent to the inferior dental nerve canal, and 3 above the inferior dental nerve canal.

**Conclusion:**

The accidental displacement of implants within the mandibular body is associated with various risk factors, including the characteristics of the bony trabeculum and the size of the medullary spaces. It is reasonable to suggest that only an adequate pre-surgical diagnostic evaluation, with the help of high-resolution tomographic images that allow a previous evaluation of these structures, will help to have better control over the other factors, thus minimizing the risk of displacement.

## Background

Complications in implant dentistry are common, but implant failure within the mandibular body is a rare occurrence that can cause significant clinical problems. Reported cases suggest a possible association with systemic conditions affecting bone metabolism [1–5]. However, there are few studies analysing the macro and micro structure of bony trabeculae, and the level at which this complication most frequently occurs is unknown. This information would be valuable in preventing such complications [1, 5–7]. Therefore, a thorough literature review is necessary to address these questions. Complications in implant dentistry can be classified in various ways, such as surgical and prosthetic complications, early and late complications, complications inherent to the technique or caused by the operator, and complications based on the stage of the osseointegration process [1, 3–7].

Excessive bleeding of the bone during the initial perforation of the bone bed is a common immediate complication of surgeries, particularly in the postero-inferior sector of the mandible [2]. This complication is associated with bone biotypes that have a thick cortical and cancellous bone with large medullary spaces. This sign could be considered as a prelude to implant failure in the mandible, as it indicates the presence of a large cavity at the level of the cancellous bone [8–12]. This complication is worsened by the loss of primary stability during dental implant placement due to over-preparation of the cortical bone bed in bones with low bone density at the cancellous level. According to Rosas et al. [12] classification, Type II-B, III-B, or Type IV bone may cause implant failure, leading to instability or, worse, displacement into anatomical spaces such as the maxillary sinus or mandibular body. Reports of implant displacement to the mandibular body describe it as an uncommon event that can cause lesions to the inferior dental nerve. This requires a new intervention for removal, which can lengthen the treatment time and potentially increase costs, negatively affecting the individual’s quality of life [1, 3–6].

The excess of torque during implant placement is the opposite of primary stability loss. This is due to a surgical protocol of under-preparation (compressive protocol) that, when associated with a very hard bone quality such as type I bone, generates microfractures in the cortical bone. These microfractures stimulate the osteoclasts, triggering significant bone remodelling and resulting in a loss of late stability of the implant within the first four weeks. This can sometimes be accompanied by prolonged pain lasting more than seven days. If this implant causes significant bone remodelling near osseous cavities, it may be absorbed into the anatomical osseous spaces [2–6].

Authors such as Christman et al. recommend the use of volumetric Cone Beam CT scans for the topographic evaluation of cortical and cancellous bone, medullary space size, and trabecular pattern [7–9]. Since 1985, bone classifications such as Lekholm and Zarb’s have been used. However, this classification is based on expert suggestions rather than controlled studies using tomographic imaging parameters. At the time of its creation, the imaging resolution was very low, which made it difficult to analyse the size of the medullary spaces and the thickness of the bony trabeculae. Additionally, this classification is subjective and does not consider the evaluation of bone hardness by the clinician [10]. Subsequently, new classifications emerged that considered the tactile sensation of bone quality, such as the Misch [8] and Norton and Gamble [11] classifications. Additionally, some classifications included the evaluation of bone biotype through Hounsfield units. Rosas et al. [12] recently presented a modification of the Lekholm and Zarb classification, which identifies six types of bone quality based on high-resolution image analysis. The bone biotype is interpreted according to the size of the medullary spaces, the thickness of the bone trabeculae, and the thickness of the bone cortex. This approach highlights the significance of the microstructure of bone trabeculae. Together with cortical thickness, bone quality plays an important role in the achievement of primary implant stability and its long-term success, which may also vary with sex, age, location of the edentulous mandibular space, and the patient’s systemic status [13].

The aim of this research was to analyse the current literature on displaced dental implants in the mandibular body, including the local and systemic variables related to their cause, and to identify the most frequent location.

## Methods

This is a retrospective and descriptive study based on a literature review conducted according to PRISMA guidelines [14]. A comprehensive search of electronic databases such as PubMed, Scopus, and Web of Science was performed until November 2022. In addition, relevant journal websites were explored. The search utilized the following MeSH terms: ‘Dental implant’, ‘displacement’, ‘dislocation’, ‘displaced’, and ‘mandible’. The search arms for each database are as follows (Table 1):

**Table 1:** Search strategy

Data base	Search strategies
Pubmed	(((dental implant) AND (dental implant[MeSH Major Topic])) AND (((displacement) OR (dislocation)) OR (displaced))) AND (mandible).
Scopus	(“dental implant”)) AND ((TITLE-ABS-KEY(displacement) OR TITLE-ABS-KEY(dislocation) OR TITLE-ABS-KEY(displaced))) AND (TITLE-ABS-KEY(mandible)).
Web of Science	(mandible) AND (displacement OR dislocation OR displaced) AND (“dental implant”).

The full title and abstract of each article were filtered by two researchers (MGG, MGL), according to inclusion and exclusion criteria:

**Inclusion criteria**:

- Studies related to bone quality, quantity or density.
- Studies with imaging methods of bone quality or density.
- Studies on displacement of dental implants in the jawbone.
- In vivo studies.
- Case reports including or not literature review.
- Studies reported in English.
- Open access articles.

**Exclusion criteria**:

- Studies with animals or dental phantoms.
- Consensus.
- Letters to the editor.
- Book chapters.
- Literature reviews.
- Local bone reaction studies (healing).
- Temporomandibular joint studies.
- Bone graft studies.
- Dental implant studies for orthodontic treatment.
- Studies of patients with any bone tumour.

All references were managed using Endnote Web (Thomson Reuters, Philadelphia, PA, USA) to eliminate duplicates. The information was then transferred to Microsoft Office Excel 2019 (Microsoft Corporation, Redmond, WA, USA) to ensure no studies were omitted. The data extraction of the selected articles (13 articles) was performed by the same researchers (MGG, MGL). Any disagreement in the extracted data was resolved by a third and fourth researcher (JRD, MEG).

## Results

**Fig. 1:**
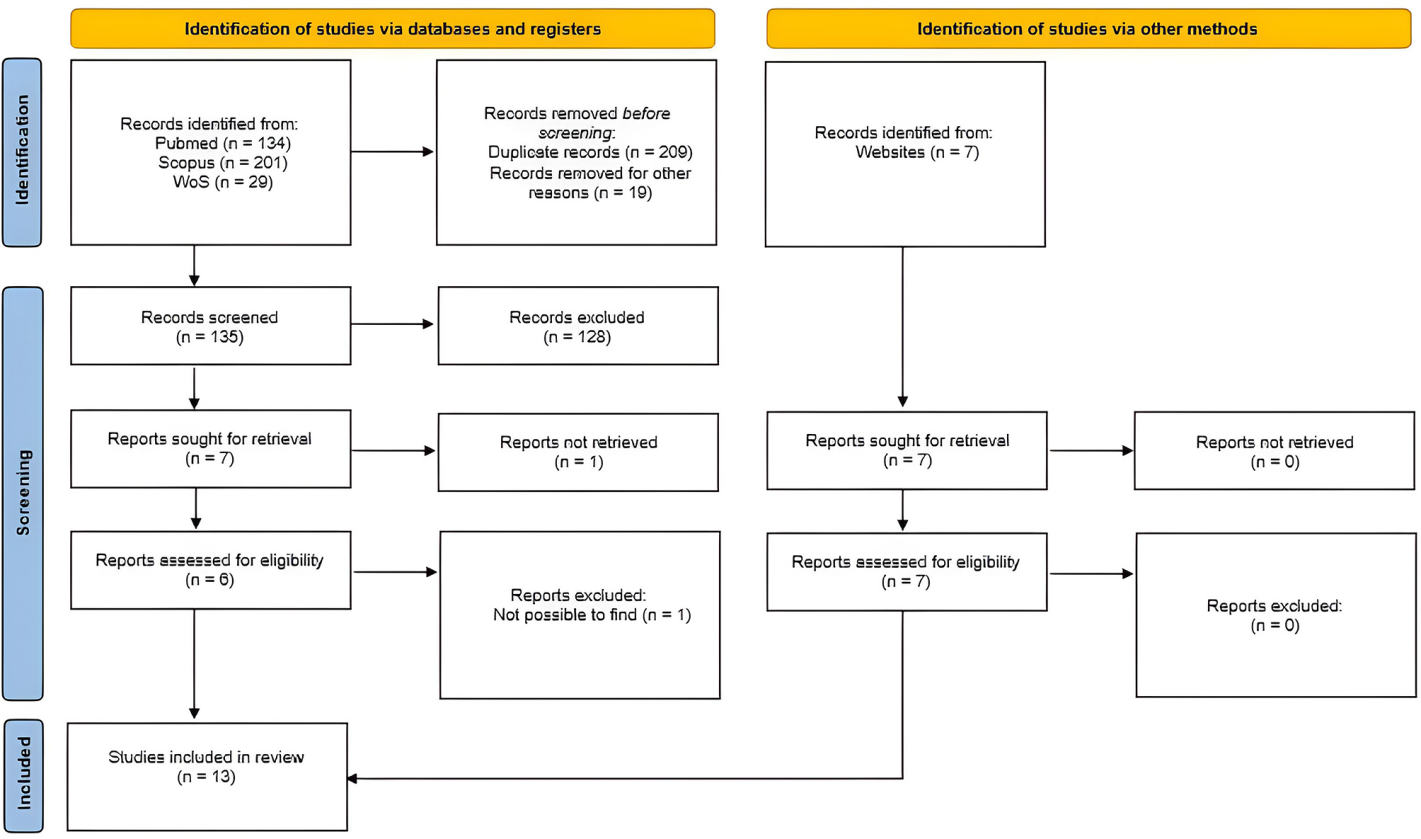
Flowchart

**Fig. 2:**
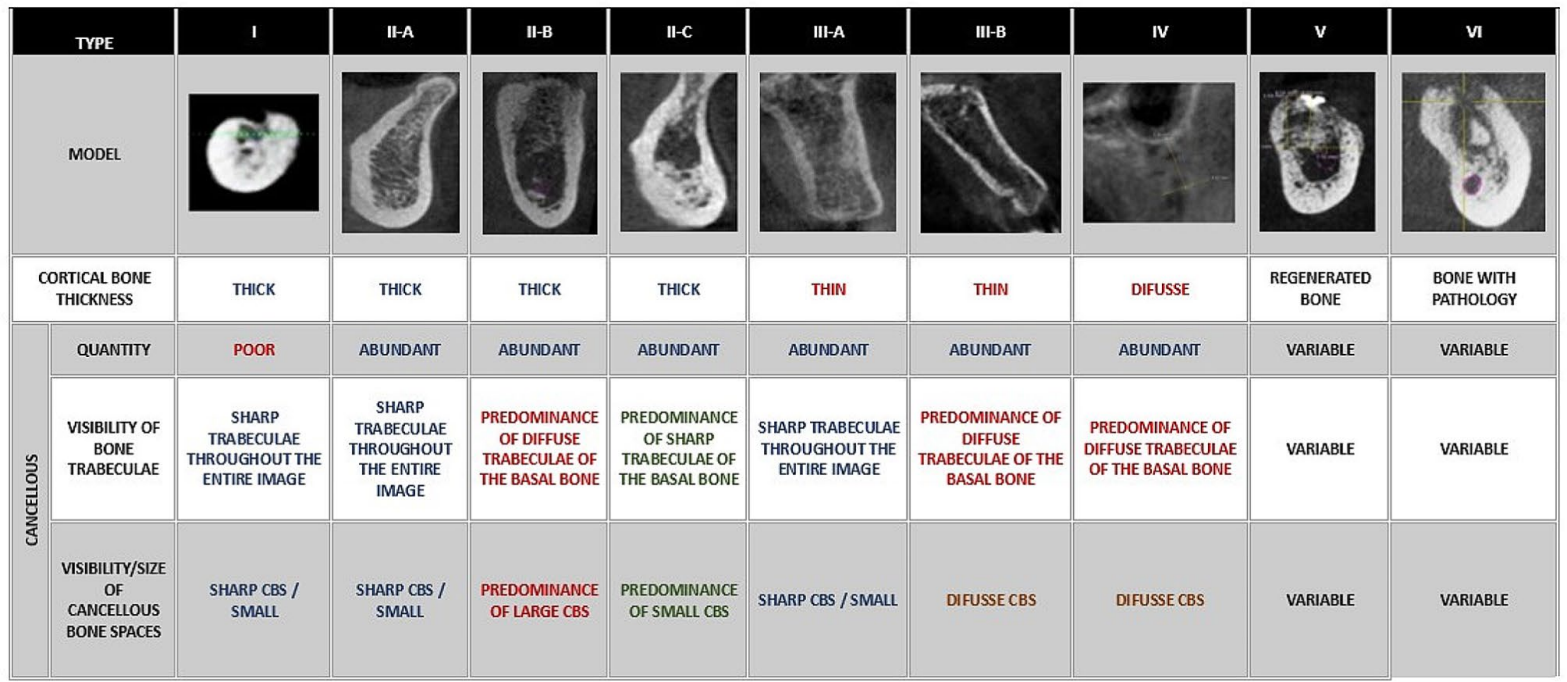
Bone quality according to the modified Lekholm and Zarb classification

**Fig. 3:**
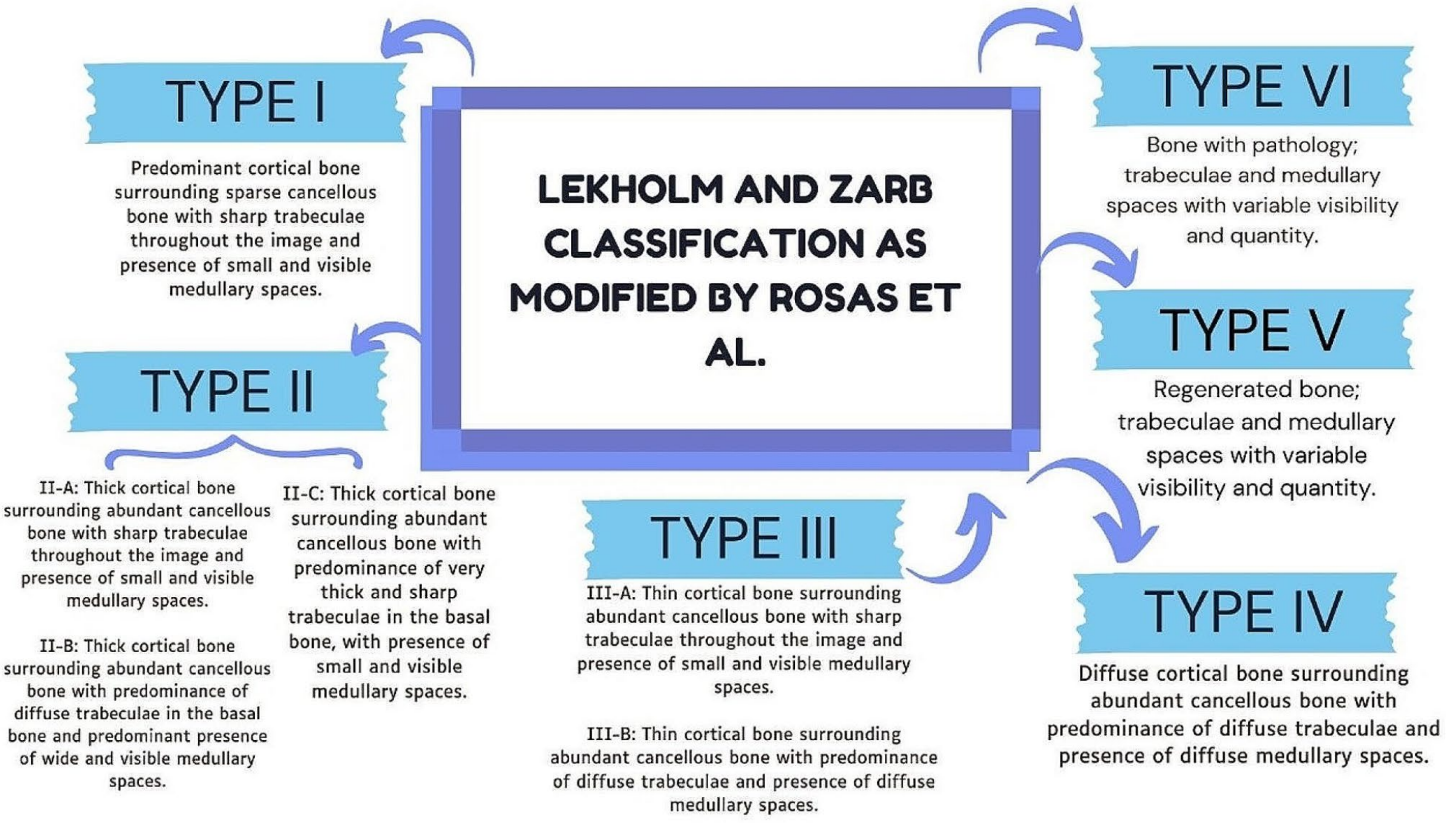
Classification and definition of bone quality according to Lekholm and Zarb as modified by Rosas et al. [12]

The literature related to the current review was accessed through Medline (134 publications), Scopus (201 publications), and Web of Science (29 publications), as well as relevant journal websites [7], resulting in a total of 371 articles. After removing duplicates and conducting a title and abstract search, thirteen articles were selected, read in full text, and included in this review (Fig. 1). To standardize data extraction and interpretation, we used the Lekholm and Zarb classification modified by Rosas et al. to define bone quality [10, 12] (Figs. 2 and 3; Table 2).

**Table 2:** Data extraction and interpretation

Number	Reference	Age (Years), Sex (M o F)	*Bone quality according to new classification	Systemic diseases	Implant location	Implant diameter x length	Direction of displacement	
							Horizontal	Vertical
1	Bayram et al. [4]	58, F	II-B	Osteoporosis	4.6	4.2 × 11.5 mm	Vestibular to the inferior dental canal	Displacement to the lower edge of the mandible
2	Doh et al. [2]	54, F	II-B	No	4.6	4.3 × 10.0 mm	Does not specify	Displacement to the middle of the mandibular body
3	Furuya et al.	72, F	III-A	No	3.6	Does not specify	Lingual to the inferior dental canal	Displacement above the inferior dental canal
4	Kim et al. [18]	68, F	III-A	No	3.6	4.1 × 10.0 mm	Lingual to the inferior dental canal	Displacement to the middle of the mandibular body
5		59, F	II-B	Hypertension and history of recurrent thyroid carcinoma.	3.6	4.0 × 8.0 mm	Vestibular to the inferior dental canal	Displacement to the lower edge of the mandible
6		34, F	II-B	Osteoporosis	4.7	4.0 × 8.0 mm	Vestibular to the inferior dental canal	Displacement to the lower edge of the mandible
7		61, F	II-B	No	4.5	4.0 × 10.0 mm	Lingual to the inferior dental canal	Displacement to the lower edge of the mandible
8		67, M	II-B	No	4.7	5.0 × 8.0 mm	Lingual to the inferior dental canal	Displacement to the lower edge of the mandible
9	Gurbanov et al. [3]	51, F	II-B	No	4.6	Does not specify	Lingual to the inferior dental canal	Displacement above the inferior dental canal
10	Kwon et al. [29]	70, F	II-B	No	4.7	4.0 × 8.0 mm	Central	Displacement to the middle of the mandibular body
11	Lee S-C et al. [1]	51, F	II-B	No	4.6	4.0 × 8.0 mm	Lingual	Displacement to the lower edge of the mandible
12		60, F	II-B	Arterial Hypertension	3.6	4.5 × 10.0 mm	Slightly to lingual	Displacement above the inferior dental canal
13		51, F	II-B	Uterine myoma	3.6	4.0 × 10.0 mm	Lingual	Displacement to the lower edge of the mandible
14	Oh J-S et al. [17]	57, F	II-B	Osteoporosis	3.6	Does not specify	Lingual to the inferior dental canal	Displacement to the lower edge of the mandible
15		67, F	II-B	Osteopenia	4.6	Does not specify	Lingual to the inferior dental canal	Displacement to the middle of the mandibular body
16	Pistilli V et al. [24]	57, F	II-A	No	4.4	3.25 × 8.5 mm	Lingual	In direct contact with the canal of the inferior dental nerve
17	Lorusso F et al. [23]	66, F	II-B	No	4.5	Does not specify	Central	Displacement to the lower edge of the mandible
18		59, F	II-B	No	3.6	4 × 11.0 mm	Slightly to lingual	Displacement to the lower edge of the mandible
19		52, F	II-B	No	3.5	Does not specify	Slightly to lingual	Displacement to the lower edge of the mandible
20	Theisen FC et al. [6]	35, F	Unclassified (no CT scans used)	No	4.6	4.0 × 10.0 mm	Does not specify	Displacement near the mandibular canal
21	Grossi-Oliveira GA, et al. [26]	59, F	Unclassified (no CT scans used)	No	4.6	4.1 × 13.0 mm	Central	Displacement to the lower edge of the mandible
22	Fakih K., Edreva M. y Stoichkov B. [15]	36, M	II-B	No	4.6 y 4.7	4.1 × 8.00 mm	Central	Displacement to the lower edge of the mandible

* Classification of bone quality according to Lekholm and Zarb as modified by Rosas et al. [12]

## Discussion

Accidental implant displacement in the medullary cavities of the posterior mandibular body has been reported in the literature [1, 5, 6]. According to Khodor et al. [15], poor bone quality and low mineral density are the main causes of this complication. They reported that the cancellous bone in the posterior mandibular sector is highly variable. Rosas et al. [10, 12] found that the medullary spaces increase in size towards the posterior sector and in a coronal direction towards the apical. This could explain why this complication was found in greater numbers in the posterior area of the mandibular body. Additionally, the systemic conditions present in the patient could increase the risk of complication.

Prior evaluation of bone quality is crucial. Therefore, it is necessary to use clear tomographies that allow for a more specific classification to be interpreted [10]. The present review utilized Rosas et al.‘s classification to reclassify cases reported as complications of fallen implants in the mandibular body [12]. It was found that most of the complications presented a type II-B bone, which is characterized by a thick cortical bone surrounding a cancellous bone with extremely wide medullary spaces.

Various factors can increase the risk of complications during implant surgery, including excessive preparation of the implant bed, systemic diseases such as osteopenia, lack of surgical experience, poor visibility due to excessive bleeding, subcrestal position implants, and the use of shorter implants near the inferior dental nerve [3, 5, 16, 17]. Among the systemic factors associated with this condition, a history of osteoporosis and osteopenia was found. These pathologies can cause not only extreme thinning of the bone trabeculae but also hyperactivity of the osteoclastic cells, resulting in greater bone resorption after the dental implant is placed. However, it cannot be stated categorically that there is a proven association between these pathologies and the failure of implant stability due to the small number of reports found. Only four of the twenty-two cases reported had a diagnosis of osteoporosis or osteopenia [1, 3, 18].

Kim et al. [18], Theisen et al. [6], and Doh et al. [5] emphasized the importance of diagnosing the so-called ‘mandibular sinus’. These authors reported that this bony defect, which can range from millimeters to centimeters, generally affects the posterior edentulous area of the mandible. It is more common in women between the ages of 40 and 60 [19, 20]. This bone defect is asymptomatic and can be observed as an unusual increase in radiolucency prior to implant placement. It can be bilateral or unilateral. The authors propose three theories to explain the etiopathogenesis of this pathology: (1) the persistence of embryonic red bone marrow, (2) the association with systemic diseases such as anaemia and osteoporosis, and (3) deficiencies in bone repair after traumatic dental extraction or early extraction of lower molars that accelerate the resorption of cancellous bone, called ‘disuse osteoporosis’ [1, 21, 22]. However, we believe that it is simply an anatomical variation of the area that may be exacerbated by systemic conditions.

Gurbanov et al. [3] and Kim et al. [18] reported that surgical inexperience of the surgeon may be associated with the displacement described above. This risk is increased by over-preparation, poor tomographic planning, excessive pressure, or low bone density of the mandibular posterior area. In this review, four cases occurred during healing cap placement [5, 6, 23], one case occurred after fixed rehabilitation [23], and the last case occurred during an attempt to remove the healing cap to reposition the implant immediately after radiographic control [24]. The use of compressive surgical protocols, along with osteotomes or bone compactors during implant placement, is believed to improve primary stability. However, excessive compression of the bone may impede osseointegration by causing significant bone resorption [11, 25]. The surgical protocol used was only specified in three cases, therefore a conclusive analysis cannot be provided [1, 5].

Bayram et al. [1] and Grossi-Oliveira et al. [26] emphasized the significance of diagnosing bone quality through radiological enhancements. Volumetric Cone Beam computed tomography is the most sensitive test for adequate bone evaluation. Of the 22 cases reported, pre-surgical planning was performed using cone beam tomographic slices in 19 cases and 2D radiographic images (panoramic) in the remaining 3 cases. The use of only 2D radiographic images may increase the risk of complications in the postero-inferior sector of the mandible [1, 6].

Primary stability is crucial for osseointegration and depends on various factors, including implant macrodesign (length and diameter), expanded platform, and surgical protocol used (over-milled - non-compressive protocol, under-milled - compressive protocol), as well as the amount of remaining bone around the implant. It is important to note that primary stability is not solely dependent on bone quality. Most of the analysed reports did not consider these data, which can be considered a serious error [27, 28]. Kwon et al. [29] have suggested inserting implants with widened neck or extended platform in patients with large medullary cavity bone, while Doh et al. [5] recommend avoiding the use of countersink or final drill in such situations to prevent implant displacement.

Kim et al. [18] reported that in 50% of cases where implants were displaced in the area of the inferior dental canal, hypoesthesia persisted after implant removal. In this review, seven patients experienced transient hypoesthesia [3, 4, 18], one patient experienced dysesthesia [17], one patient experienced paresthesia [24], and one patient experienced severe pain [6] as complications. However, in most cases, the displaced implants were resolved by removal. Only in two cases was the implant unable to be removed.

**Fig. 4:**
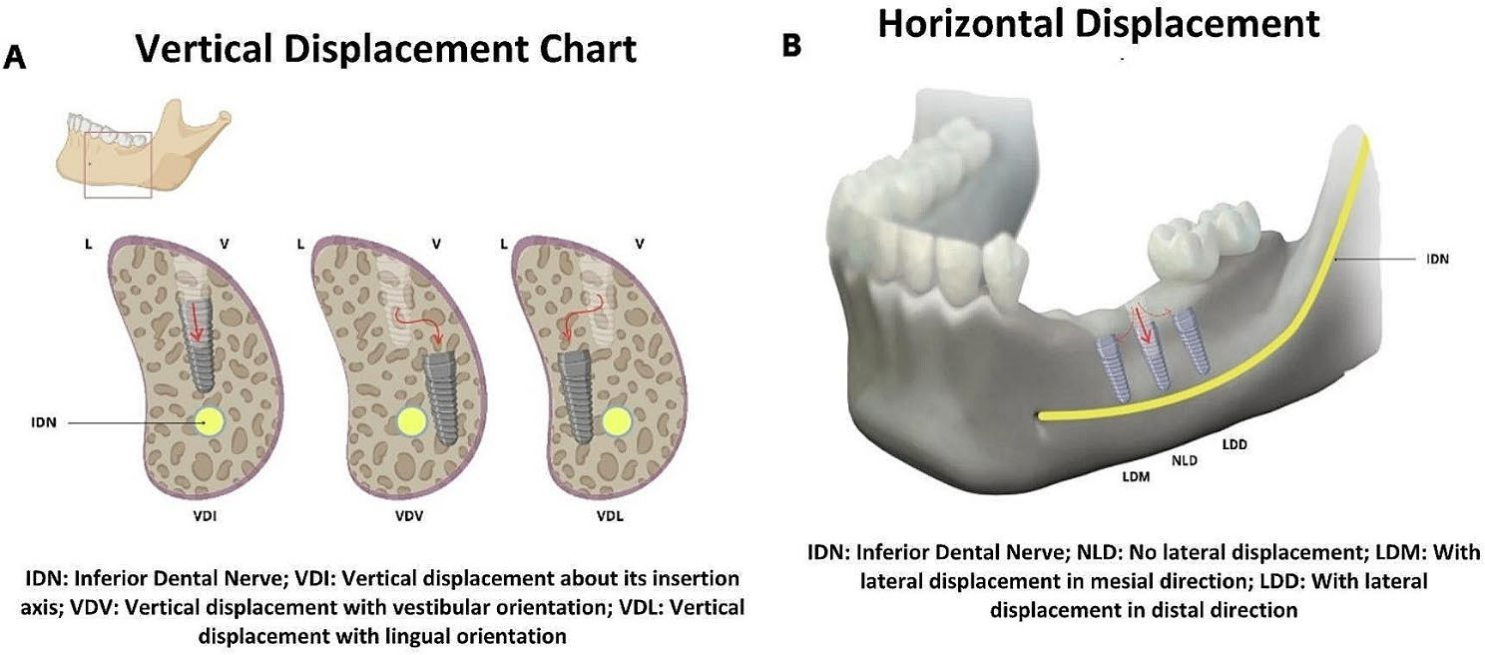
Graph showing horizontal and vertical displacement of the dental implant in relation to the inferior dental canal. **A**) Vertical displacement of the implant. **B**) Horizontal displacement of the implant

Khodor et al. [15] and Furuya et al. [30] concluded that if there are no infections or sensitive complications, the implant can remain in its displaced position in the bone for osseointegration. However, this may result in a more complex and highly invasive procedure in the future, with a potential risk of damage to the inferior dental nerve. In contrast, Scarano et al. [23] concluded that a displaced implant generates infection at the site, causing inflammation due to foreign body reaction, which is even more severe when the migration is toward the sublingual space, causing pain, trismus, and swelling. In such cases, immediate removal of the implant is necessary. Removal of a displaced dental implant is generally a safe surgical procedure. However, according to reports, there is a higher incidence of hypoesthesia in cases where implants are displaced lingually in the inferior dental canal, due to the difficulty of removal [18]. Two types of approaches can be used: crestal and lateral. Crestal is the simplest but has a limited operative field of vision. The lateral approach, on the other hand, offers a sufficient operative field that guarantees the management of displaced implants with a greater enlargement of the working area and is recommended in cases where the implant is located lingually under the mandibular canal [18]. Of the reports found, 22 cases presented horizontal displacement. Four were displaced towards the vestibular [1, 18], fourteen towards the lingual [3, 4, 17, 18, 23, 24, 30], and four remained in the center [23, 29]. In 24 cases of vertical displacement, 12 were displaced towards the inferior border of the mandible [1, 4, 17, 18, 23, 26], 9 to the middle [5, 6, 17, 18, 24, 29] or next to the inferior dental nerve canal, and 3 were above this canal [3, 4, 30] (Fig. 4). The majority of cases were resolved with a lateral approach using piezoelectric and osteotomes for removal, followed by filling with biomaterials. In terms of the most common location of complications, 14 cases were found at the level of the first molar, 4 at the level of the second molar, 3 at the level of the second premolar, and 1 at the level of the first premolar.

In this literature review, the main limitations that increase the risk of complications are inadequate diagnosis of bone quality, lack of appropriate choice of compressive surgical protocol, and inadequate selection of implant macro-design for deficient bone quality. In cases of type II-B bone quality with extremely wide medullary spaces, it is recommended to transform the bone biotype to a type V (regenerated bone). To achieve this, a biomaterial is placed in the bone defect before inserting the dental implant. The implant can be placed immediately or deferred until the sixth month.

## Conclusion

The accidental displacement of implants within the mandibular body is associated with various risk factors, including the characteristics of the bony trabeculum and the size of the medullary spaces. It is reasonable to suggest that only an adequate pre-surgical diagnostic evaluation, with the help of high-resolution tomographic images that allow a previous evaluation of these structures, will help to have better control over the other factors, thus minimizing the risk of displacement.

## Data Availability

All data generated or analysed during this study are included in this published article. All included articles in this research are available from josecarlos.rosas@upsjb.edu.pe on reasonable request.

## Abbreviations

PRISMA: Preferred Reporting Items for Systematic reviews and Meta-Analyses
CT: Computerised tomography
